# Association between Early Pregnancy Maternal Folate and Glycemic Indices at Oral Glucose Tolerance Test: A Systematic Review and Meta-analysis

**DOI:** 10.1016/j.cdnut.2025.107531

**Published:** 2025-08-23

**Authors:** Nishanthi Periyathambi, Swetha Sampathkumar, Nithya Sukumar, Yonas Ghebremichael-Weldeselassie, Ponnusamy Saravanan

**Affiliations:** 1 Department of Populations, Evidence and Technologies, Warwick Applied Health, Warwick Medical School, Gibbet Hill, University of Warwick, Coventry, United Kingdom; 2 Department of Diabetes, Endocrinology and Metabolism, George Eliot Hospital NHS-Trust, Nuneaton, United Kingdom; 3 Centre for Development, Evaluation, Complexity, and Implementation in Public Health Improvement (DECIPHer), School of Social Science, Cardiff University, Glamorgan Building, King Edward VII, Cardiff, United Kingdom; 4 School of Mathematics and Statistics, The Open University, Milton Keynes, United Kingdom; 5 Head of Department of Populations, Evidence and Technologies, Warwick Centre for Global Health, Warwick Medical School, University of Warwick, Coventry, United Kingdom

**Keywords:** folate, maternal nutrition, gestational diabetes, glycemic indices, pregnancy glycemic, control, systematic review and meta-analysis

## Abstract

**Background:**

Folate plays a crucial role in fetal development, but its relationship with maternal glucose metabolism remains inconclusive. Recent meta-analyses have suggested a correlation between high folate and risk of gestational diabetes mellitus in pregnancy; however, its association with different glycemic parameters has not yet been explored.

**Objectives:**

This study aims to comprehensively synthesize evidence and test the association between early pregnancy circulating folate (<16 wk of gestation) and glycemic indices measured during oral glucose tolerance testing (OGTT) at 24–28 wk.

**Methods:**

We conducted a systematic search of databases up to 25 June, 2025, examining the relationship between early pregnancy folate and maternal glycemic indices. Study quality was assessed by Newcastle-Ottawa Scale. Standardized effect sizes (std. β coefficients) for serum/plasma folate were pooled using a random-effects model. Subgroup and sensitivity analyses were performed to account for between-study heterogeneity.

**Results:**

Ten studies were included in this meta-analysis. Early pregnancy maternal folate levels were positively associated with glycemic indices measured at the time of OGTT. One standard deviation (nmol/L) increase in early pregnancy serum folate was associated with 0.01 [95% confidence interval (CI): −0.001, 0.01] mmol/L higher fasting, 0.17 (95% CI: 0.15, 0.18) mmol/L higher 1-h glucose, and 0.10 (95% CI: 0.05, 0.15) mmol/L higher 2-h glucose levels during OGTT Subgroup analyses revealed similar positive association between mid-pregnancy circulating folate and glucose levels measured at the time of OGTT despite substantial between-study heterogeneity (*I*^*2*^>70%).

**Conclusions:**

Our analysis suggests a possible association between higher early pregnancy folate levels and higher glucose levels at the time of OGTT. However, these findings should be interpreted cautiously, given the methodological limitations and the limited number of studies included in this review.

This trial was registered at PROSPERO as CRD42021255022.

## Introduction

Gestational diabetes mellitus (GDM), characterized by glucose intolerance first identified during pregnancy, affects ∼14% of all pregnancies worldwide [1,2]. GDM is associated with an increased risk of adverse maternal and neonatal outcomes, including large-for-gestational age infants, shoulder dystocia, preterm delivery, cesarean section, neonatal hypoglycemia, and neonatal intensive care unit admissions [3]. Given these potential complications, early identification of modifiable risk factors is crucial for mitigating these adverse outcomes. Recently, many observational studies have identified a significant association between the vitamins involved in 1-carbon metabolism (such as folate and B12) and risk of GDM in pregnancy [[4], [5], [6]]. Animal studies in rats have demonstrated a protective relationship of folate status with improved β-cell function and reduced fasting blood glucose levels [7]. Furthermore, a meta-analysis of folic acid supplementation has shown to improve glycemic control in the nonpregnant population; however, its relationship with GDM risk remains complex and inconclusive [4,8]. Although animal studies offer valuable mechanistic hypotheses on the association between folate and glucose metabolism, these pathways cannot be directly extrapolated to human studies.

Moreover, although folic acid supplementation during pregnancy is essential for preventing neural tube defects and other congenital anomalies, emerging evidence suggests that an excess folate level may be associated with increased risk of GDM [[9], [10], [11]]. Furthermore, excessive folate in the presence of B12 deficiency may exacerbate hyperglycemia in pregnancy [4,9,12]. Although many studies have examined the association between folate and GDM risk, relatively few have elucidated the complex interplay between folate, β-cell function, insulin secretion, and glucose metabolism [[13], [14], [15]]. Current systematic reviews and meta-analyses indicate a possible increased risk of GDM with elevated prenatal folate levels, although the evidence remains inconclusive [5,[16], [17], [18]]. A significant limitation of the existing reviews and meta-analyses is their primary focus on maternal folate on GDM diagnosis and the lack of detailed data on individual glycemic indices. This approach limits the ability to fully understand the complex interaction between folate and fasting and postload glucose levels [5,17].

To address these knowledge gaps, we aimed to systematically review the literature on the association between early pregnancy circulating folate levels and individual glycemic indices measured during the oral glucose tolerance test (OGTT). In this systematic review and meta-analyses, we aimed to synthesize and understand the role of folate in gestational glucose homeostasis and its potential association with maternal glucose intolerance.

## Methods

This study was conducted in accordance with the PRISMA guidelines for reporting systematic reviews and meta-analyses [19]. The study protocol was registered in PROSPERO (registry: CRD42021255022 and available on https://www.crd.york.ac.uk/prospero/display_record.php?ID=CRD42021255022) on 14 May, 2021 and last updated on 25 June, 2025 to extend the search period.

### Search strategy and selection criteria

A systematic search of the literatures was performed in Medline, Embase, Web of Science core collection, and Cochrane library database from inception until 25 June, 2025, using the combination of text and MeSH heading search strategy with the following terms: “folate,” “glycemic indices,” and “pregnancy.” The details of search terms and retrieved articles were presented in Supplemental Table 1. Furthermore, we searched the reference lists of eligible reports and Google Scholar for other potentially relevant studies. Prospective and retrospective cohort studies were included if effect estimates or equivalents that examined the association between circulating maternal serum and/ or plasma folate levels and glycemic indices in pregnancy were reported. Studies were excluded if they had not reported either fasting glucose, 1-h or 2-h post glucose levels measured at the time of OGTT, studies that did not provide information on circulating serum/plasma folate levels, studies that have reported only folic acid supplementation or intervention in pregnancy, or were done in population that consisted of pregnant women with pre-existing diabetes or other underlying chronic conditions and not published in English language (Supplemental Table 2).

The study selection was performed by 2 independent reviewers (NP and SS) using the Rayyan (http://rayyan.qcri.org) web application [20]. Studies were screened based on title and abstracts, when duplicate reports from the same study were identified, only the publication with the most relevant information was included. Eligible articles following abstract screening were assessed by 2 reviewers, and any disagreement was resolved with a third reviewer (NS and/or PS).

### Data extraction and statistical analysis

The primary outcomes of this systematic review were the associations between maternal circulating folate levels in early pregnancy and glycemic indices measured during OGTT including fasting plasma glucose, 1-h postload plasma glucose, and 2-h postload plasma glucose. The secondary outcomes were the associations between maternal circulating folate levels measured concurrently with OGTT. The exposure variable was maternal circulating folate concentrations measured either in early pregnancy (<16 wk of gestation ) or at the time of OGTT (typically 24–28 wk of gestation). We have defined early pregnancy as <16 wk of gestation due to clinical constraints, as many women do not receive their first antenatal blood work until between 12 and 16 wk—particularly in healthcare systems with later booking practices [4]. All folate measurements (serum and/or plasma) were standardized to SI units (nmol/L) before analysis. If a study reported plasma folate concentration, we used a conversion factor of 1.3 to serum folate equivalents [21]. For the meta-analysis, we included studies that reported regression coefficients (standardized or unstandardized) or correlation coefficients examining these associations, either unadjusted or adjusted for key confounding variables including age, BMI, and ethnicity. The effect size of interest was the standardized regression coefficient, which represents the SD change in the outcome (glycemic indices) per SD increase in the exposure (early pregnancy folate concentration).

Newcastle-Ottawa Scale (NOS) was used to evaluate risk of bias in studies, including domains of selection of study groups, comparability, and the ascertainment of outcome of interest [22]. The risk of bias results is presented in Supplemental Table 3, highlighting the study quality and domain-specific assessment of included studies. For each study, we extracted author name, publication year, region, study design, number of participants, mean age, mean BMI, family history of diabetes, gestational age at the time of folate measurement, mean folate levels, and an effect size with 95% confidence interval (CI) of glycemic indices. Folate quantification reported in conventional units was converted into SI units (nmol/L) before data transformations in the meta-analysis. Studies that reported estimates with log-transformed variables were transformed back to the normal scale using Rodríguez-Barranco et al. [23] method based on the mean (SD) values of the predictor variable. After standardization of effect estimates using Nieminen et al. [24], we generated pooled estimates across studies using random-effects meta-analysis. Detailed information on the data transformations and the effect sizes utilized in this meta-analysis are presented in Supplemental Table 4. Adjusted standardized effect estimates after controlling for potential confounders reported in the original studies were included in the meta-analysis as they are methodologically robust. Pooled estimates of unadjusted effect sizes were reported in the Supplemental Figures 1–3. We also systematically assessed each study's potential for residual confounding by evaluating adjustment for the key variables included in each study that were known to influence both folate status and glucose metabolism. In this meta-analysis, 2 studies were not included due to the lack of mean glucose levels to estimate standardized effect size; however, they were included in the narrative synthesis [25,26]. Furthermore, the association between maternal folate and subsequent OGTT glycemic indices was analyzed as a longitudinal association, acknowledging the original cohort design of all included studies. Any discrepancies in data extraction and synthesis were consulted with a statistician (YWG) before data analysis. Inverse variance of the standardized regression coefficient was used to weight the studies based on an estimate of statistical size [27].

The *I*^*2*^ statistic was used to calculate the percentage of variability across studies due to between-study heterogeneity. For the *I*^*2*^ statistic, values of < 25%, 25%–50%, 50%–75%, and >75% were classified as low, moderate, high, and very high between-study heterogeneity, respectively. To identify potential sources of heterogeneity, random-effects metaregression analyses were used to evaluate if variations in study regions, gestational week of folate quantification, or risk of bias contributed to the observed heterogeneity between studies. Furthermore, we conducted a sensitivity analysis by separately pooling studies that originally measured serum folate and those that measured plasma folate to understand the different exposure measurement on the effect size (standardized regression coefficient). To evaluate the impact of individual studies on the overall effect size, we performed a priori sensitivity analysis by recalculating the overall effect size after sequentially excluding each study. We conducted parallel meta-analyses using both unadjusted and adjusted effect estimates to assess the impact of confounding. Additional analyses included stratification by confounding risk level and metaregression with confounding score as a predictor variable. Funnel plots were used to investigate publication bias by plotting the natural logarithm of the effect sizes against their SEs following Egger’s test. The meta-analysis was performed using R programming language version 4.2.2 (https://www.R-project.org/). A *P* value of <0.05 was considered as statistically significant.

## Results

**FIGURE 1:**
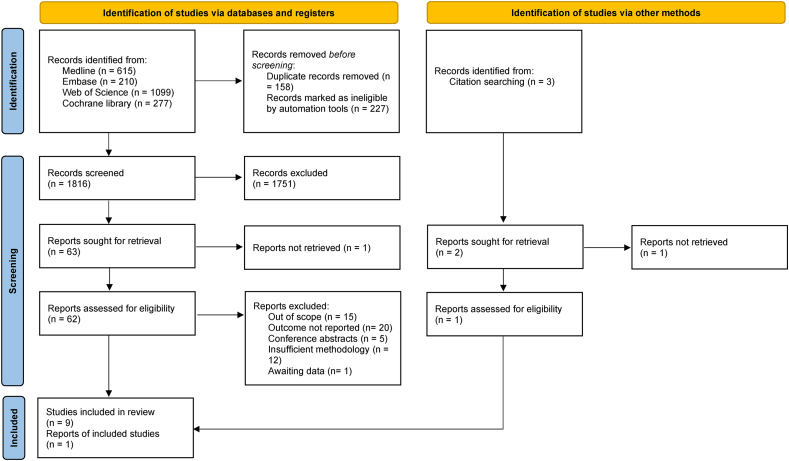
PRISMA chart for study selection. Flow diagram of study selection process for the systematic review and meta-analysis. This PRISMA flow diagram outlines the study selection process.

The systematic search of databases identified 2201 articles, and 2 additional articles were identified by manual search. Of these, 385 were duplicates and 1816 were excluded after title and abstract screening. In total, 63 articles qualified for full-text assessment; of these, 53 were excluded for lack of glycemic indices measurements. Details regarding the exclusion criteria and specific reasons for excluding each article are presented in Supplemental Table 2. Finally, 10 articles were included in the systematic review and 7 of them were included in the meta-analysis [4,10,12,[28], [29], [30], [31],^32^]. We attempted to contact the authors for additional data; raw data were received only from Saravanan et al. [4] during the review period. The flow diagram of the study selection is provided in Figure 1.

Table 1 [26,31,32] shows the study characteristics. Among the 10 studies, 5 were prospective cohort studies [4,10,25,29,31], 2 were retrospective cohort study [26,28], 2 were cross-sectional studies [12,31], 1 was a secondary analysis of the control group in a randomized controlled trial [30], and 1 was a retrospective case-control study [32]. The study quality was assessed using the NOS scale, with a score of ≥ 7 considered high quality (Supplemental Table 3). Of the 10 studies included in this review, 2 had a score of 9 [4,30], 3 had a score of 8 [10,25,29], 2 had a score of 7 [26,28], and 2 studies had a score <7 with low quality [3,12,31] (Supplemental Table 3). Regarding the folate quantification, 9 studies had reported serum folate levels [4,10,12,26,[28], [29], [30], [31], [32]], whereas plasma folate was reported in 1 study [25]. Only 3 studies reported circulating folate levels in early pregnancy, whereas the remaining studies measured folate at the time of the OGTT [4,26,28]. Circulating folate levels were measured at a gestational age of 9–15.7 wk for early pregnancy and 24–28 wk for late gestation, coinciding with the timing of the OGTT.

**TABLE 1:** Study characteristics.

Author	Country	Setting cohort	Study participants	Age (y)	BMI (kg/m^2^)	Gestational age of folate measurement	Mean folate	Folate assay	Reported regression coefficients	Maximum covariate adjustment available
Cheng et al. (2022) [32]	China	Retrospective case-control study	744	—	—	24–28 wk	—	Serum folate	Fasting: OR= Q1 1.82 (0.93, 3.55) Q2 1.73 (0.89, 3.37) Q3 Ref Q4 1.92 (0.98, 3.74) 1-h: OR= 1.14 (0.43, 3.08) 0.60 (0.20, 1.82) Ref 1.43 (0.54, 3.78) 2-h: OR 0.49 (0.20, 1.18) 0.77 (0.35, 1.66) Ref 0.88 (0.41, 1.88)	Age, BMI, gravidity, family history of diabetes
Lai et al. (2018) [10]	Singapore	Prospective cohort study	913	30.60	—	24–28 wk	35.23 nmol/L	Serum folate	FPG: −0.03 (−0.06, 0.001) in unadjusted model and −0.02 (−0.06, 0.01), −0.02 (−0.05, 0.02) in models 1 and 2, respectively 1-h: — 2-h: 0.26 (0.16, 0.35) in unadjusted model and 0.17 (0.08, 0.27), 0.15 (0.04, 0.25) in model 1 and 2, respectively	Age, ethnicity, education, parity, BMI, serum B12 and B6 and family history of diabetes
Li et al. (2019) [12]	China	Cross-sectional study	406	29.40	—	24–28 wk	9.02 ng/mL	Serum folate	FPG: 0.04 (−0.03, 0.11) in unadjusted model and 0.05 (−0.02, 0.13) in model 1, and 0.08 (0.01, 0.16) in model 2 1-h: 0.60 (0.30, 0.91) in unadjusted model and 0.49 (0.19, 0.80) in model 1, and 0.51 (0.19, 0.83) in model 2 2-h: 0.46 (0.22, 0.69) in unadjusted model and 0.44 (0.19, 0.68) in model 1, and 0.45 (0.19, 0.71) in model 2	Age, ethnicity, BMI, education, parity, family history of diabetes
Liu et al. (2022) [5]	China	Retrospective cohort study	42,478	30.30	21.90	15.7 wk	42.29 nmol/L	Serum folate	FPG: 0.00 (−0.00, 0.01) in unadjusted model and 0.01 (0.00, 0.01) in model 1 1-h: 0.17 (0.15, 0.18) in unadjusted model and 0.15 (0.13, 0.17) in model 1 2-h: 0.14 (0.12, 0.15) in unadjusted model and 0.12 (0.11, 0.13) in model 1	Age, BMI, fetal gender, parity, education
Looman et al. (2019) [25]	Netherlands	Prospective cohort study	105	31.90	25.00	24–26 wk	29.7 nmol/L	Plasma folate	FPG: −0.003 (−0.007, 0.002) 1-h: Not clear as OGTT was done in <12 wk and at 24 wk of gestation 2-h: −0.002 (−0.013, 0.008)	Age, ethnicity, BMI, parity, GDM history and gestational age at measurement
Saravanan et al. (2021) [4]	United Kingdom	Prospective cohort study	4746	30.51	30.80	12.5 wk	35.9 nmol/L	Serum folate	FPG: only quadratic regression was reported 1-h: not reported 2-h: 0.08 (0.04, 0.13) in model 1	Age, BMI, parity, family history of diabetes, household income, smoking, homocysteine
Van Weelden et al. (2021) [30]	United Kingdom	Secondary analysis of RCT	959	30.70	35.20	24–28 wk	9.60 μg/L	Serum folate	FPG: unadjusted model showed 0.002 (−0.005, 0.009), 0.0005 (−0.007, 0.008) in model 1 and −0.003 (−0.008, 0.007) in model 2 and 0.0007 (−0.007, 0.008) in model 3 1-h: 0.046 (0.018, 0.074) in unadjusted model, 0.033 (0.003, 0.063) in model 1, 0.029 (0.001, 0.059) in model 2 and 0.031 (0.001, 0.061) 2-h: 0.017 (0.003, 0.037) in unadjusted model, 0.011 (−0.01, 0.033) in model 1, 0.009 (−0.012, 0.030) in model 2 and 0.011 (−0.010, 0.033)	Age, ethnicity, BMI, parity, smoking, education, deprivation index, GDM history, family history of diabetes
Wang et al. (2022) [2]	China	Prospective cohort study	1065	30.80	—	24–28 wk	11.8 nmol/L	Serum folate	FPG: Pearson correlation *r* = −0.010 1-h: *r* = 0.025 2-h: *r* = 0.012	Unadjusted correlation
Wang et al. (2023) [31]	China	Cross-sectional study	1254	29.80	22.57	24–28 wk	9.41 ng/mL	Serum folate	FPG: 0.05 (0.01, 0.09) in unadjusted model, 0.07 (0.03, 0.12) in model 1 1-h: 0.44 (0.29, 0.59) in unadjusted model and 0.32 (0.16, 0.48) in model 1 2-h: 0.35 (0.23, 0.47) in unadjusted model and 0.29 (0.15, 0.42) in model 1	Age, BMI, education, smoking, drinking, family history of diabetes, parity, rs1801131 genotypes, and homocysteine
Zhang et al. (2024) [26]	China	Retrospective cohort study	27,128	30.70	21.10	9–13 wk	Pooled estimate: 28.3 nmol/L	Serum folate	FPG: FA quartiles > 32.5 nmol/L: Q3: 4.56 (4.55–4.57) Q4: 4.62 (4.61–4.63) 1-h: — 2-h: —	Age, BMI, education, parity, and family history of GDM

This table summarizes key details of the included studies, including study setting, sample size, maternal characteristics such as age and BMI, study design, folate measurement methods, gestational age at the time of folate quantification, reported regression coefficient as a measure of effect estimate and the maximum covariates adjusted in each study.

Abbreviations: FA, folic acid; FPG, fasting plasma glucose; GDM, gestational diabetes mellitus; OR, odds ratio; Q1, quartile 1; Q2, quartile 2; Q3, quartile 3; Q4, quartile 4; RCT, randomized control trial.

### Maternal folate and fasting glucose

**FIGURE 2:**
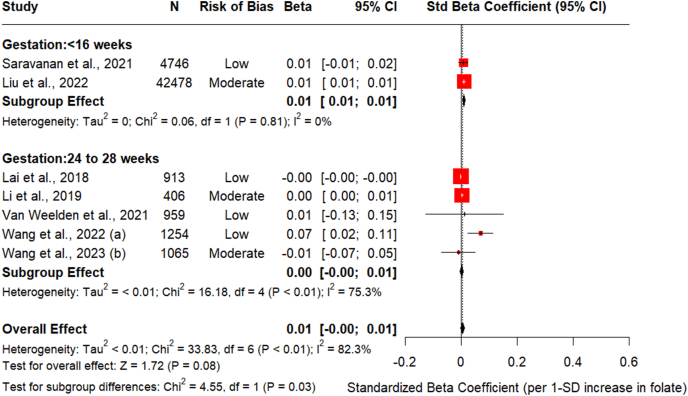
Association between maternal circulating folate and fasting glucose at OGTT. Forest plot to assess the association between circulating folate and fasting glucose (mmol/L) for 1SD (1 nmol/L) change in maternal folate stratified by the timing of folate measurement (including early pregnancy at < 16 wk of pregnancy and mid-pregnancy circulating folate concentrations measured between 24 and 28 wk of pregnancy). Regression coefficients were adjusted for key maternal covariates in all the studies except for Wang et al. (2022) which reported unadjusted correlation between circulating folate and fasting plasma glucose. Data are reported as standardized β-coefficients and standardized SE with 95% CI. Pooled estimates of unadjusted effect estimates were reported in Supplemental Figure 1. CI, confidence interval; OGTT, oral glucose tolerance test; Std. beta, standardized β-coefficients; Std. SE, standardized SE.

To understand the association between serum/plasma folate and fasting glucose measured at the time of OGTT, 7 studies were included in the meta-analysis [4,10,12,[28], [29], [30], [31]]. The relationship between early pregnancy folate and fasting glucose in a random-effects model showed that 1 SD nmol/L increase in folate was positively associated with 0.01 mmol/L higher glucose levels at the time of OGTT [std. β = 0.01 (95% CI: 0.005, 0.02), *I*^2^ = 0%, *P* value < 0.001] (Figure 2). However, no significant association between folate and fasting glucose levels was identified when folate levels were measured at the time of OGTT [std. β = 0.002 (95% CI: −0.003, 0.007), *I*^2^ =75.3%, *P* value < 0.001]. Sensitivity analysis using leave-one-out analysis revealed that removing Lai et al. [10] from the model (folate measured at the time of OGTT) reduced heterogeneity with an overall effect size of std. β = 0.01 (95% CI: 0.002, 0.01), *I*^2^ =58.5%, *P* value = 0.009 (Supplemental Figure 4). Metaregression analysis revealed a significant negative relationship between confounding adjustment level and effect size (β_1_ = −0.0034 mmol/L per additional confounder, 95% CI: −0.005, −0.002, *P* value < 0.0001). This indicates that studies with more comprehensive confounder adjustment report systematically smaller effect sizes, suggesting the presence of positive confounding bias in unadjusted analyses. The confounding score explained 99.93% of between-study heterogeneity, demonstrating that differential adjustment across studies is the primary source of variation in our meta-analysis (Table 2 and Supplemental Figure 1). Due to an insufficient number of studies, Egger’s test to assess potential publication bias could not be performed. However, we have included a funnel plot, and any conclusions drawn from it should be interpreted with caution (Supplemental Figure 5).

**TABLE 2:** Metaregression analysis to access the heterogeneity due to covariates.

Meta-analysis	Number of studies	β_1_ (95% CI)	*P* value	*R*^2^ (%)	*I*^2^ residual (%)	Interpretation
Fasting plasma glucose	7	−0.003 (−0.005, −0.002)	<0.001	99.93	3.93	Strong evidence of positive confounding bias. Each additional confounder reduces effect by 0.0034 mmol/L. Confounding explains ∼99.9% of heterogeneity.
1-h plasma glucose	5	−0.003 (−0.068, 0.063)	0.94	0.00	98.82	No evidence of confounding bias. Association robust to adjustment differences. Heterogeneity might be driven by other factors.
2-h plasma glucose	7	−0.053 (−0.104, −0.002)	0.04	34.74	99.34	Moderate evidence of positive confounding bias. Each additional confounder reduces effect by 0.053 mmol/L. Confounding partially explains heterogeneity.

This table shows the metaregression analyses outcomes conducted using restricted maximum likelihood estimation. The confounding score was calculated as the total number of confounders adjusted for in each study (range: 0–10), with higher scores indicating more comprehensive confounder adjustment.

Abbreviations: *β*_1_, change in effect size per additional confounder adjusted; *R*^2^, percentage of heterogeneity explained by confounding score; *I*^2^ residual, remaining unexplained heterogeneity after accounting for confounding.

### Maternal folate and 1-h glucose

**FIGURE 3:**
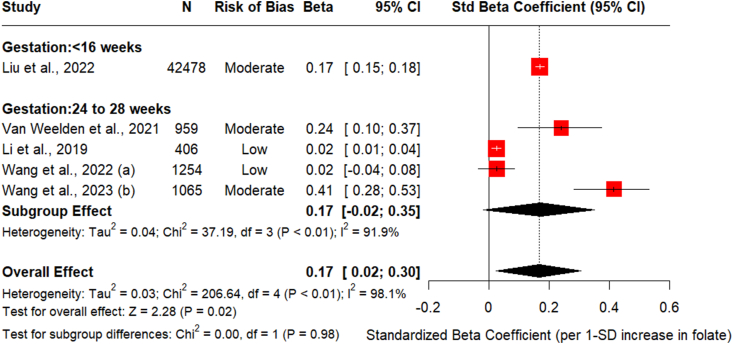
Association between maternal circulating folate and 1-h post glucose load at OGTT. Forest plot to assess the association between circulating folate and 1-h glucose (mmol/L) for 1SD (1 nmol/L) change in maternal folate stratified by the timing of folate measurement (including early pregnancy at <16 wk of pregnancy and mid-pregnancy circulating folate concentrations measured between 24 and 28 wk of pregnancy). Regression coefficients were adjusted for key maternal covariates in all studies except Wang et al. (2022) which reported unadjusted correlation between serum folate and 1-h plasma glucose, respectively. Data are reported as standardized β-coefficients and standardized SE with 95% CI. 1-h glucose, 1-h plasma glucose; CI, confidence interval; OGTT, oral glucose tolerance test; Std. beta, standardized β-coefficients; Std. SE, standardized SE.

Overall, 5 studies presented the relationship between maternal circulating folate (early and at the time of OGTT) and 1-h glucose levels measured at the time of mid-gestational OGTT [12,[28], [29], [30], [31]]. The pooled estimate on the association between early pregnancy folate and 1-h glucose showed that 1 SD nmol/L in increase circulating folate was associated with 0.17 mmol/L increase in 1-h glucose measured at the time of OGTT [std. β = 0.17 (95% CI: 0.15, 0.18, *I*^2^ =Na, *P* value < 0.001)] (Figure 3). Subgroup analysis indicated that higher heterogeneity was not due to the timing of folate measurement, where the association between early pregnancy folate and 1-h glucose showed no between-study heterogeneity (*P* value = 0.98). Sensitivity analysis showed that omitting Li et al. [12] from the model significantly reduced the between-study heterogeneity from 97.7% to 91.5%% with an overall improvement in the effect estimate of 0.21 mmol/L [std. β = 0.21 (95% CI: 0.04, 0.36, *I*^2^ = 91.5%, *P* value = 0.001)] increase in 1-h glucose for 1 SD increase in folate levels. (Supplemental Figure 6). Metaregression analysis found no significant relationship between confounding adjustment level and effect size (β_1_ = −0.003 mmol/L per additional confounder, 95% CI: −0.068, 0.063, *P* value = 0.94). The confounding score explained 0% of between-study heterogeneity (*R*^2^ = 0.00%), indicating that differential confounder adjustment across studies does not systematically bias the pooled estimate. This supports the validity of our approach to pool studies with varying adjustment levels, as the folate-glucose association appears robust to confounding adjustment. The substantial remaining heterogeneity (*I*^2^ = 98.8%) suggests other study characteristics, rather than confounding adjustment patterns, are the primary drivers of between-study variation (Table 2 and Supplemental Figure 2)

Due to an insufficient number of studies, Egger’s test to assess potential publication bias could not be performed (Supplemental Figure 7).

### Maternal folate and 2-h glucose

**FIGURE 4:**
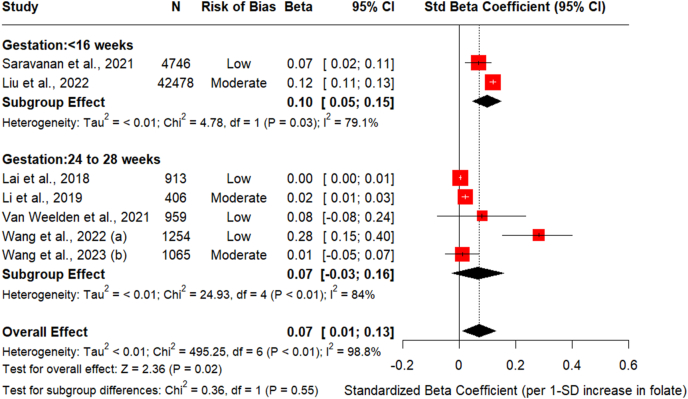
Association between maternal circulating folate and 2-h post glucose load at OGTT. Forest plot to assess the association between circulating folate and 2-h glucose (mmol/L) for 1SD (1 nmol/L) change in maternal folate stratified by the timing of folate measurement (including early pregnancy at <16 weeks of pregnancy and mid-pregnancy circulating folate concentrations measured between 24 and 28 weeks of pregnancy). Regression coefficients were adjusted for key maternal covariates in all studies except Wang et al. (2022) which reported unadjusted correlation between serum folate and 2-h plasma glucose respectively. Data are reported as standardised b-coefficients and standardized β-coefficients and standardized SE with 95% CI. 2-h glucose, 2-h plasma glucose; CI, confidence interval; OGTT, oral glucose tolerance test; Std. beta, standardized β-coefficients; Std. SE, standardized SE.

Figure 4 presents the pooled effect size of 7 studies that investigated the association between maternal circulating folate (early at <16 wk of gestation and at the time of OGTT) and 2-h post blood glucose at the time of OGTT done between 24 and 28 wk of gestation [4,10,12,[28], [29], [30], [31]], where 3 studies reported early pregnancy circulating folate levels [4,26,28]. 1 SD increase in early pregnancy folate was associated with 0.10 mmol/L higher 2-h glucose [std. β = 0.10 (95% CI: 0.05, 0.15), *I*^2^ = 79.1% , *P* value =0.01) from the pooled estimate of 2 studies [4,28]. Subgroup analysis found no difference between the timing of folate measurement (*P* value = 0.56. Sensitivity analysis showed that omitting Liu et al. [28] from the model slightly improved the between-study heterogeneity with *I*^2^ =84,4%%, with the effect estimate reduced to std. β = 0.06 [(95% CI: −0.006, 0.08, *I*^2^ = 84.4%, *P* value = 0.08)] (Supplemental Figure 8). Metaregression analysis revealed a significant negative relationship between confounding adjustment level and effect size (β_1_ = −0.0530 mmol/L per additional confounder, 95% CI: −0.1038, −0.0023, *P* = 0.041). This indicates that studies with more comprehensive confounder adjustment report systematically smaller effect sizes, suggesting the presence of positive confounding bias in less-adjusted analyses. The confounding score explained 34.74% of between-study heterogeneity, demonstrating that differential adjustment across studies is a substantial source of variation in our meta-analysis. Although significant residual heterogeneity remains (*I*^2^ = 99.34%), this analysis quantifies the systematic bias from inadequate confounding control and supports reporting stratified results by adjustment level or prioritizing more comprehensively adjusted estimates in interpretation (Table 2 and Supplemental Figure 3). Due to an insufficient number of studies, Egger’s test to assess potential publication bias could not be performed (Supplemental Figure 9).

## Discussion

In this systematic review, we investigated the relationship between early pregnancy folate levels (mean gestational age = 14.1 wk), and mid-pregnancy glycemic indices assessed during OGTT. Our findings reveal a possible positive association between early pregnancy folate levels and subsequent glycemic parameters. Specifically, 1 SD (nmol/L) increase in early pregnancy folate was associated with 0.01 mmol/L of higher fasting glucose, 0.17 mmol/L of higher 1-h glucose, and 0.10 mmol/L of higher 2-h glucose levels during OGTT. Similar weak positive associations were seen between folate levels and 1-h and 2-h glucose levels when folate was measured at the time of OGTT. However, it is important to note that heterogeneity among studies and the limited number of available studies reduced the overall certainty of evidence for these associations. Therefore, any conclusions drawn from this analysis should be interpreted with caution.

The pathophysiology of GDM has been widely studied, with particular focus on early pregnancy modifiable risk factors such as obesity, excessive weight gain, diet, nutrition, and physical inactivity [33]. Although prenatal folic acid supplementation is crucial for preventing neural tube defects, concerns have emerged regarding elevated levels of unmetabolized folate in pregnant women [34]. Our findings suggest that elevated folate levels could precede the development of impaired glucose tolerance in pregnancy, contributing to the growing body of evidence linking early pregnancy folate status to GDM risk. Recent systematic reviews have indicated an association between prenatal folic acid intake, circulating folate levels, and an increased GDM risk, although meta-analyses have yielded inconclusive results [18]. Notably, periconceptional folate status, particularly elevated serum and red blood cell folate levels in the first trimester, has been associated with increased GDM risk [5,17].

Our results align with previous meta-analyses that reported associations between higher prenatal folate levels and increased GDM risk [5,17,35]. In addition, our study provides a more nuanced understanding by examining the relationship between early pregnancy folate status and specific glycemic parameters, rather than just on GDM diagnosis. Unlike earlier meta-analyses, our findings partially support the hypothesis that higher early pregnancy folate might be involved in impaired insulin secretion. Interestingly, although folic acid supplementation has been shown to lower fasting glucose and insulin resistance in nonpregnant populations, albeit at a smaller magnitude, our results indicate that early pregnancy folate may be associated with higher fasting glucose in pregnancy [8]. The underlying mechanisms for this association remain unclear. However, Selhub et al. demonstrated that excess folate can lead to increased total homocysteine levels. Elevated homocysteine may inhibit the insulin receptor signaling, thereby reducing protein kinase B (Akt) phosphorylation and resulting in the dysregulation of insulin-stimulated glycogen synthesis [36,37], which in turn can increase fasting glucose levels.

We have demonstrated that gestational folate resulted in higher 1-h and 2-h glucose levels at OGTT, irrespective of the timing of folate measurement. This elevated peripheral insulin resistance due to excess folate may be explained by the presence of unmetabolized folate. Unmetabolized folate reduces methylation potential leading to homocysteine accumulation, which may impair pancreatic β-cell function, insulin secretion, and can lead to mitochondrial dysfunction [38,39]. These mitochondrial abnormalities contribute to impaired muscle ATP production and glucose uptake [[39], [40], [41]]. Furthermore, a few studies identified that disruption of 1-carbon cycle metabolites may induce insulin resistance and GDM in pregnancy [40,41]. Supporting these findings, 2 Indian studies showed that maternal high-folate in the presence of low-vitamin B12 status resulted in higher adiposity and insulin resistance in offspring [42,43]. This association can be explained by the “folate trap” phenomenon, which can occur in the presence of vitamin B12 deficiency. The folate trap disrupts fatty acid oxidation, mitochondrial functional integrity, and increases endoplasmic reticulum stress, ultimately leading to insulin resistance [36,44]. However, our systematic review did not assess vitamin B12 status alongside folate measurement, representing an important limitation as the interaction between these 2 nutrients may significantly influence metabolic outcomes.

The underlying mechanisms for the observed associations remain to be understood completely. Periconceptional folic acid supplementation has been positively correlated with elevated levels of unmetabolized folate, which may play a significant role in GDM pathogenesis [34,45]. This unmetabolized folate has been implicated in immune dysregulation, specifically through the reduction of natural killer cell cytotoxicity, and triggering the development of GDM [[46], [47], [48]]. In a multiethnic United Kingdom cohort study including 4746 pregnant women, Saravanan et al. showed that more than one-third of women have supraphysiological (2–3 times the SD) folate levels at the end of the first trimester of pregnancy, which is of no value in protecting neural tube defects (NTDs) beyond the first 28 d of gestation [4,49]. Excess folate can potentially mask vitamin B12 deficiency, thereby, exacerbating insulin resistance via altered adipose-derived circulating microRNAs [36,50]. Altogether, these findings suggest that the association between high maternal folate status and impaired glucose tolerance is complex and may involve multiple glucose-insulin signaling pathways.

Our review and meta-analyses have several strengths. This meta-analysis represents the first comprehensive synthesis investigating the association between early pregnancy circulating folate levels and various glycemic indices measured at the time of OGTT. The analyses mainly included high-quality studies, as evidenced by NOS scores ≥ 7, thereby enhancing the certainty of the evidence. Moreover, the pooled estimates were presented as standardized effect sizes of folate reported in each study, with adjustments made for key confounding variables, including maternal age, BMI, and family history of diabetes. To strengthen our findings and account for potential heterogeneity, we conducted rigorous sensitivity analyses, which allowed us to identify robust associations. However, we acknowledge several limitations in our study. Primarily, the number of studies with reported early pregnancy folate and glycemic indices at the time of OGTT in late pregnancy was small (*n* = 4) and primarily from China and United Kingdom. This limits our generalizability and reduces the statistical power, evident by the wide CIs, to detect potential biases or sources of heterogeneity [51]. Second, our review comprised studies with wide prepregnancy BMI distributions, a notable limitation given that higher BMI has been shown to be associated with functional folate deficiency due to altered folate metabolism [52]. Third, the paucity of studies precluded the use of metaregression to elucidate the influence of BMI as a potential source of bias, which reinforces the need for cautious interpretation of our findings. Finally, limitations of our systematic review included the inability to examine the differential effects of folic acid supplementation duration and the timing of folate measurement (fasting compared with nonfasting) on glycemic parameters at OGTT. Nevertheless, it is important to note that plasma or serum folate serves as an indicator of recent folate intake and is substantially influenced by folic acid supplementation.

In conclusion, this systematic review and meta-analysis suggests a weak positive association between early pregnancy folate levels and subsequent glycemic indices measured during OGTT, especially the 1- and 2-h glucose levels. As more than a third of pregnant women can have supraphysiological folate levels, with increasing prevalence of GDM, our findings call for studies to understand the complex relationship between folate and glycemia in pregnancy. We strongly emphasize that our findings should not undermine the established public health recommendation of prepregnancy folic acid supplementation for neural tube defect prevention. Additionally, investigating the potential underlying mechanisms and studies focusing on long-term metabolic outcomes of offspring exposed to varying maternal folate levels may provide valuable insights into intergenerational health implications.

## Author contributions

The authors’ responsibilities were as follows – NP, SS: carried out the screenings and reviews; NP, YG-W: carried out the analysis of the articles; NP, NS, PS: drafted and revised the manuscript; and all authors: read and approved the final manuscript.

## Data availability

Only publicly published data were used in this review. Data transformation described in the manuscript, R scripts and codes, and excel codes will be made available on request to the corresponding author.

## Funding

NP was funded by Chancellor’s International Scholarship to pursue PhD by the University of Warwick, and SS was funded by the Novo-Nordisk International Doctoral Training Scholarship to pursue a PhD. PS, NS, and YG-W were part-funded by Medical Research Council (MRC), United Kingdom (MR/J000094/1). All authors declare that the supporting sources had no such involvement or restrictions regarding publication of this manuscript.

## Conflict of interest

The authors report no conflict of interest.
