# Supplementary material for: Association between Early Pregnancy Maternal Folate and Glycemic Indices at Oral Glucose Tolerance Test: A Systematic Review and Meta-analysis
Source: Curr Dev Nutr. 2025 Aug 23;9(9):107531. doi: 10.1016/j.cdnut.2025.107531 (PMC12478247; doi:10.1016/j.cdnut.2025.107531)
Supplement: Multimedia component 1 [file mmc1.docx]

Supplementary Files

## Supplementary Table 1: The medical database and MeSH terms used to search relevant publications on the association between gestational folate levels and individual glycaemic indices during pregnancy

| Database | Search Component | Search Terms | Results |
| --- | --- | --- | --- |
| MEDLINE (Ovid) | Folate Exposure | folate.mp. or exp Folic Acid/ | 60,044 |
|  |  | folic acid.mp. or exp Folic Acid/ | 60,381 |
|  |  | exp Folic Acid/ or periconception.mp. or exp Preconception Care/ | 46,664 |
|  |  | exp Folic Acid Deficiency/ or exp Folic Acid/ or folate supplement.mp. | 45,994 |
|  |  | vitamin b9.mp. or exp Folic Acid/ | 44,149 |
|  |  | tetrahydrofolate.mp. or exp Tetrahydrofolates/ | 21,652 |
|  |  | exp Hyperhomocysteinemia/ or exp Folic Acid/ or methylene tetrahydrofolate.mp. | 49,869 |
|  |  | Combined Folate Terms (OR) | 85,804 |
|  | Diabetes/Glucose Outcomes | gestational diabetes.mp. or exp Diabetes, Gestational/ | 30,127 |
|  |  | exp Pregnancy in Diabetics/ or exp Diabetes, Gestational/ or exp Glucose Tolerance Test/ or GDM.mp. | 66,865 |
|  |  | exp Pregnancy in Diabetics/ or exp Diabetes, Gestational/ or diabetes in pregnancy.mp. | 30,268 |
|  |  | hyper glycemia.mp. or exp Blood Glucose/ or exp Hyperglycemia/ | 219,869 |
|  |  | oral glucose tolerance.mp. or exp Glucose Tolerance Test/ | 47,754 |
|  |  | OGTT.mp. or exp Glucose Tolerance Test/ | 42,608 |
|  |  | Combined Diabetes Terms (OR) | 271,359 |
|  | Pregnancy Context | exp Pregnancy/ or exp Pregnancy Trimester, Third/ or exp Pregnancy in Diabetics/ or pregnancy.mp. or exp Pregnancy Trimester, First/ or exp Pregnancy Trimester, Second/ or exp Pregnancy Outcome/ | 1,175,401 |
|  |  | gestation.mp. or exp Pregnancy/ | 1,105,520 |
|  |  | exp Pregnancy/di [Diagnosis] | 1,068 |
|  |  | periconception.mp. or exp Preconception Care/ | 3,332 |
|  |  | Combined Pregnancy Terms (OR) | 1,210,152 |
|  | Final Results | Combined (AND) + Filters | 615 |
| Embase | Folate Exposure | folate.mp. or exp folic acid/ | 95,994 |
|  |  | exp folic acid deficiency/ or exp folic acid/ or folicacid.mp. or exp vitamin/ | 829,999 |
|  |  | exp folic acid/ or folate supplement.mp. | 80,645 |
|  |  | exp homocysteine/ or exp folic acid deficiency/ or exp cyanocobalamin/ or exp folic acid/ or vitamin b9.mp. or exp hyperhomocysteinemia/ | 140,340 |
|  |  | tetrahydrofolate.mp. or exp tetrahydrofolic acid/ | 4,802 |
|  |  | methylene tetrahydrofolate.mp. or exp methylenetetrahydrofolic acid/ | 2,079 |
|  |  | Combined Folate Terms (OR) | 868,282 |
|  | Diabetes/Glucose Outcomes | gestational diabetes.mp. or exp pregnancy diabetes mellitus/ | 63,255 |
|  |  | exp diabetes mellitus/ or exp pregnancy diabetes mellitus/ or GDM.mp. | 1,439,496 |
|  |  | Pregnancy in Diabetics.mp. or exp pregnancy diabetes mellitus/ | 59,435 |
|  |  | exp oral glucose tolerance test/ or glucose.mp. or exp impaired glucose tolerance/ or exp continuous glucose monitoring system/ or exp glucose blood level/ or exp glucose tolerance/ or exp glucose tolerance test/ or exp glucose/ or exp blood glucose monitoring/ or exp glucose intolerance/ or exp glucose level/ or exp intravenous glucose tolerance test/ | 1,148,601 |
|  |  | exp hyperglycemia/ or hyperglycemia.mp. | 163,978 |
|  |  | glucose tolerance.mp. or exp glucose tolerance/ | 144,686 |
|  |  | glucose intolerance.mp. or exp glucose intolerance/ | 29,874 |
|  |  | exp glucose tolerance test/ or exp oral glucose tolerance test/ or exp glucose blood level/ or exp diabetes mellitus/ or oral glucose tolerance.mp. | 1,620,448 |
|  |  | OGTT.mp. or exp oral glucose tolerance test/ | 52,440 |
|  |  | Combined Diabetes Terms (OR) | 2,193,669 |
|  | Pregnancy Context | exp second trimester pregnancy/ or exp pregnancy test/ or exp third trimester pregnancy/ or exp first trimester pregnancy/ or exp pregnancy outcome/ or pregnancy.mp. or exp pregnancy/ | 1,108,778 |
|  |  | gestation.mp. or exp pregnancy/ | 938,108 |
|  |  | exp prenatal exposure/ or exp prenatal care/ or prenatal.mp. | 360,779 |
|  |  | periconception.mp. or exp pregnancy/ or exp conception/ | 861,704 |
|  |  | preconception.mp. or exp maternal care/ or exp prepregnancy care/ or exp pregnancy outcome/ or exp pregnancy/ | 933,907 |
|  |  | Combined Pregnancy Terms (OR) | 1,349,771 |
|  | Final Results | Combined (AND) + Filters | 210 |
| Web of Science | Folate Exposure | folate OR "folic acid" OR "vitamin b9" OR tetrahydrofolate* OR "methylene tetrahydrofolate" OR "5-methyltetrahydrofolate" OR "5-MTHF" OR "folate supplement*" OR "folic acid deficiency" OR "folate deficiency" OR "folate metabolite*" OR "unmetabolized folic acid" OR "UMFA" | 74,806 |
|  |  | periconception* OR preconception* OR "preconception care" OR "periconceptional supplementation" | 14,559 |
|  |  | Combined Folate Terms (OR) | 87,081 |
|  | Diabetes/Glucose Outcomes | "gestational diabetes" OR "gestational diabetes mellitus" OR GDM OR "diabetes in pregnancy" OR "pregnancy diabetes" OR "diabetes mellitus in pregnancy" | 36,218 |
|  |  | "glucose tolerance test" OR OGTT OR "oral glucose tolerance" OR hyperglycemia OR hyperglycaemia OR "blood glucose" OR "fasting glucose" OR "glucose intolerance" OR glycemia OR glycaemia | 251,904 |
|  |  | Combined Diabetes Terms (OR) | 278,037 |
|  | Pregnancy Context | pregnan* OR gestation* OR "maternal health" OR "antenatal care" OR "prenatal care" OR trimester OR "first trimester" OR "second trimester" OR "third trimester" | 912,517 |
|  | Final Results | Combined (AND) + Filters | 1,099 |
| Cochrane Library | Folate Exposure | Folic acid | 5,841 |
|  |  | folate or "folic acid" or "vitamin b9" or tetrahydrofolate* or "5-methyltetrahydrofolate" or "folate supplement*" or periconception* or preconception* | 7,987 |
|  |  | Combined Folate Terms (OR) | 8,000 |
|  | Diabetes/Glucose Outcomes | Gestational Diabetes | 5,174 |
|  |  | Glucose Tolerance Test | 8,990 |
|  |  | "gestational diabetes" or GDM or "glucose tolerance test" or OGTT or hyperglycemia or "blood glucose" | 55,192 |
|  |  | Combined Diabetes Terms (OR) | 57,076 |
|  | Pregnancy Context | Pregnancy | 83,309 |
|  |  | pregnan* or gestation* or trimester | 108,912 |
|  |  | Combined Pregnancy Terms (OR) | 108,912 |
|  | Final Results | Combined (AND) | 277 |
| TOTAL UNIQUE ARTICLES |  |  | 2,201 |

## Supplementary Table 2: Studies Excluded from the Systematic Review

| Author, Year | Study Title | Reason for Exclusion | Comment |
| --- | --- | --- | --- |
| Krishnaveni, G.V., et al. (2009) | Low plasma vitamin B12 in pregnancy is associated with gestational 'diabesity' and later diabetes | Inadequate outcome measures | Did not report glycemic indices at time of OGTT |
| Yuan, X., et al. (2022) | Association of folate and vitamin B12 imbalance with adverse pregnancy outcomes among 11,549 pregnant women: An observational cohort study | Inappropriate exposure | Did not report folate measurement |
| Sobczyńska-Malefora, A., et al. (2022) | Vitamin B12 and Folate Markers Are Associated with Insulin Resistance During the Third Trimester of Pregnancy in South Asian Women, Living in the United Kingdom, with Gestational Diabetes and Normal Glucose Tolerance | Inadequate outcome measures | Did not report glycemic indices at time of OGTT |
| Xie, K., et al. (2019) | Association of maternal folate status in the second trimester of pregnancy with the risk of gestational diabetes mellitus | Inadequate outcome measures | Did not report glycemic indices at time of OGTT |
| Jankovic-Karasoulos, T., et al. (2021) | Maternal folate, one-carbon metabolism and pregnancy outcomes | Inadequate outcome measures | Did not report glycemic indices at time of OGTT |
| Tarim, E., et al. (2004) | Elevated plasma homocysteine levels in gestational diabetes mellitus | Inadequate outcome measures | Did not report glycemic indices at time of OGTT |
| Barzilay, E., et al. (2018) | Fetal one-carbon nutrient concentrations may be affected by gestational diabetes | Inappropriate population | Folate measured in fetus |
| Berglund, S.K., et al. (2016) | Maternal, fetal and perinatal alterations associated with obesity, overweight and gestational diabetes: An observational cohort study (PREOBE) | Non-relevant exposure and outcome | Did not report folate and glycemic indices at OGTT |
| Guven, M.A., et al. (2006) | Elevated second trimester serum homocysteine levels in women with gestational diabetes mellitus | Inadequate outcome measures | Did not report glycemic indices at time of OGTT |
| Chen, et al. (2022) | Association of Folic Acid Supplementation in Early Pregnancy with Risk of Gestational Diabetes Mellitus: A Longitudinal Study | Inadequate exposure measures | Did not report circulating folate levels |
| Cheng, et al. (2019) | The Associations between the Duration of Folic Acid Supplementation, Gestational Diabetes Mellitus, and Adverse Birth Outcomes based on a Birth Cohort | Inadequate exposure measures | Did not report circulating folate levels |
| Chibireva, et al. (2023) | Association of maternal folate and B12 vitamin status with gestational diabetes mellitus: Still an open issue | Inadequate outcome measures | Did not report glycemic indices at time of OGTT |
| Madiwale, et al. (2025) | Longitudinal assessment of maternal micronutrients (folate and vitamin B12) and homocysteine levels in women who develop gestational diabetes mellitus | Inadequate outcome measures | Did not report glycemic indices at time of OGTT |
| Liu, et al. (2025) | Associations of maternal serum folate, vitamin B12 and their imbalance with gestational diabetes mellitus: The mediation effects of the methionine cycle related metabolites | Inadequate outcome measures | Did not report glycemic indices at time of OGTT |
| Zheng, et al. (2024) | Gestational diabetes mellitus is associated with distinct folate-related metabolites in early and mid-pregnancy: A prospective cohort study | Inadequate outcome measures | Awaiting data request |
| Tranidou, et al. (2024) | Impact of Maternal Micronutrient Intake on Gestational Diabetes Risk: Results from Greece's BORN2020 Prospective Cohort Study | Inadequate outcome measures | Did not report glycemic indices at time of OGTT |
| Yong, et al. (2020) | The association between dietary patterns before and in early pregnancy and the risk of gestational diabetes mellitus (GDM): Data from the Malaysian SECOST cohort | Inadequate outcome measures | Did not report glycemic indices at time of OGTT |
| Wen, et al. (2018) | Effect of high dose folic acid supplementation in pregnancy on pre-eclampsia (FACT): double blind, phase III, randomised controlled, international, multicentre trial | No outcomes reported- study withdrawn | No outcomes and withdrawn study |
| Godfrey, et al. (2023) | Maternal B-vitamin and vitamin D status before, during, and after pregnancy and the influence of supplementation preconception and during pregnancy: Prespecified secondary analysis of the NiPPeR double-blind randomized controlled trial | Inadequate outcome measures | Did not report glycemic indices at time of OGTT |
| Chen, et al. (2021) | Association of Maternal Folate and Vitamin B12 in Early Pregnancy With Gestational Diabetes Mellitus: A Prospective Cohort Study | Only RBC folate measurement was reported | Did not report serum/plasma folate measurement |
| Blesson, C.S., et al. (2018) | Folate treatment partially reverses gestational low-protein diet-induced glucose intolerance and the magnitude of reversal is age and sex dependent | Out of scope- No population of interest | Out of scope |
| Cui, Y.F., et al. (2021) | Association of maternal pre-pregnancy dietary intake with adverse maternal and neonatal outcomes: A systematic review and meta-analysis of prospective studies | Inappropriate study design | Meta-analysis |
| Donovan, S., et al. (2020) | Folic Acid from Fortified Foods and/or Supplements during Pregnancy and Lactation and Health Outcomes: A Systematic Review | Inappropriate study design | Meta-analysis |
| Ermumcu, M.S.K., et al. (2023) | Effects of High-dose Folic Acid Supplementation on Maternal/Child Health Outcomes: Gestational Diabetes Mellitus in Pregnancy and Insulin Resistance in Offspring | Inadequate outcome measures | Out of scope |
| Guo, Y.F., et al. (2022) | Folic Acid Supplementation in Early Pregnancy, Homocysteine Concentration, and Risk of Gestational Diabetes Mellitus | Inadequate outcome measures | Out of scope |
| Hammouda, S.A.I., et al. (2019) | Reduced serum concentrations of vitamin B12 and folate and elevated thyroid-stimulating hormone and homocysteine levels in first-trimester pregnant Saudi women with high A1C concentrations | Inadequate outcome measures | Out of scope |
| He, J., et al. (2022) | Vitamin B12 status and folic acid/vitamin B12 related to the risk of gestational diabetes mellitus in pregnancy: a systematic review and meta-analysis of observational studies | Inappropriate study design | Meta-analysis |
| Shakya, S., et al. (2022) | Association between low serum Vitamin B12 and high folate level in pregnant women with gestational diabetes mellitus | Conference abstract | Insufficient data for analysis |
| Yang, Y., et al. (2021) | Association between maternal folate status and gestational diabetes mellitus | Inappropriate study design | Case-control study |
| Liu, X.H., et al. (2022) | The association between serum folate and gestational diabetes mellitus: a large retrospective cohort study in Chinese population | Inappropriate study design | Retrospective design |
| Sultana, N., et al. (2011) | Association of gestational diabetes mellitus with dietary intake of macro- and micro-nutrients | Conference abstract | Insufficient data for analysis |
| Zhu, L.Y., et al. (2023) | Association of Folic Acid Supplementation, Dietary Folate Intake and Serum Folate Levels with Risk of Gestational Diabetes Mellitus: A Matched Case-Control Study | Inappropriate study design | Case-control study |
| Maas, V.Y.F., et al. (2021) | Associations between periconceptional lifestyle behaviours and adverse pregnancy outcomes | Inappropriate exposure | Lifestyle behaviors not specific folate measurements |
| Khin, M.O.O., et al. (2015) | Correlation of Serum Folate Levels with Glucose Levels in Gestational Diabetes Mellitus (GDM) in Relation to Vitamin B12 Levels | Conference abstract | Insufficient data for analysis |
| Huang, L.L., et al. (2019) | Duration of periconceptional folic acid supplementation and risk of gestational diabetes mellitus | Inappropriate exposure | Folic acid supplementation duration, not serum levels |
| Yang, Z., et al. (2023) | Effect of folic acid supplementation in the association between short sleep duration and gestational diabetes mellitus | Inappropriate exposure | Folic acid supplementation as modifier, not primary exposure |
| Li, N.N., et al. (2022) | Effects of maternal folate and vitamin B12 on gestational diabetes mellitus: a dose-response meta-analysis of observational studies | Inappropriate study design | Meta-analysis |
| Zhu, B.B., et al. (2016) | Folic Acid Supplement Intake in Early Pregnancy Increases Risk of Gestational Diabetes Mellitus: Evidence From a Prospective Cohort Study | Inappropriate exposure | Folic acid supplementation, not serum levels |
| Petry, C.J., et al. (2021) | Folic acid supplementation during pregnancy and associations with offspring size at birth and adiposity: a cohort study | Inadequate outcome measures | Did not assess GDM or glucose levels |
| Li, M.Y., et al. (2023) | Joint effect of maternal pre-pregnancy body mass index and folic acid supplements on gestational diabetes mellitus risk: a prospective cohort study | Inappropriate exposure | Folic acid supplementation, not serum levels |
| Zhang, Q., et al. (2021) | Joint effect of urinary arsenic species and serum one-carbon metabolism nutrients on gestational diabetes mellitus: A cross-sectional study of Chinese pregnant women | Inappropriate study design | Cross-sectional design |
| Li, S., et al. (2019) | Joint effects of folate and vitamin B12 imbalance with maternal characteristics on gestational diabetes mellitus | Inappropriate study design | Cross-sectional design |
| Krishnaveni, G.V., et al. (2007) | Maternal B12, folate during pregnancy: relationships with gestational diabetes, offspring size and glucose/insulin concentrations | Conference abstract | Insufficient data for analysis |
| Lambert, V., et al. (2023) | Maternal dietary components in the development of gestational diabetes mellitus: a systematic review of observational studies to timely promotion of health | Inappropriate study design | Systematic review |
| Machairiotis, N., et al. (2021) | Nutrients that modulate gestational diabetes mellitus: A systematic review of cohort studies Jan 2019-Jan 2020 | Inappropriate study design | Systematic review |
| Liu, Q.Y., et al. (2022) | Periconceptional folate and gestational diabetes mellitus: a systematic review and meta-analysis of cohort studies | Inappropriate study design | Meta-analysis |
| Li, M.Y., et al. (2019) | Prepregnancy Habitual Intakes of Total, Supplemental, and Food Folate and Risk of Gestational Diabetes Mellitus: A Prospective Cohort Study | Inappropriate exposure | Dietary folate intake, not serum levels |
| Maher, A., et al. (2021) | The Relationship Between Folate, Vitamin B12 and Gestational Diabetes Mellitus With Proposed Mechanisms and Foetal Implications | Inappropriate study design | Narrative review |
| Hong, S.C., et al. (2011) | The relationship between folic acid supplementation and serum folate level in early pregnancy and pregnancy outcomes: MOCEH (Mothers and Children's Environmental Health) study | Conference abstract | Insufficient data for analysis |
| Khin, M.O., et al. (2016) | Role of maternal serum folate and vitamin B12 on glycaemia and birth weight in gestational diabetes (GDM) | Conference abstract | Insufficient data for analysis |
| Wang, Y.G., et al. (2023) | Serum folate mediates the associations of MTHFR rs1801133 polymorphism with blood glucose levels and gestational diabetes mellitus in Chinese Han pregnant women | Inappropriate exposure | Genetic polymorphism as primary exposure- Related study was included |
| Segura, M.T., et al. (2010) | Study of the folate status throughout pregnancy in obese women and women with gestational diabetes | Conference abstract | Insufficient data for analysis |
| Zou, J.M., et al. (2023) | U-shaped Association Between Folic Acid Supplementation and the Risk of Gestational Diabetes Mellitus in Chinese Women | Inappropriate exposure | Folic acid supplementation, not serum levels |
| Wang, L., et al. (2021) | Vitamin B12 and Folate Levels During Pregnancy and Risk of Gestational Diabetes Mellitus: A Systematic Review and Meta-Analysis | Inappropriate study design | Meta-analysis |

## Supplementary Table 3: Newcastle-Ottawa Scale (NOS) for assessing the quality of studies (Risk of Bias)

| **Author, Year** | **Selection** | | | | **Comparability** | **Outcome** | | | **Total score** |
| --- | --- | --- | --- | --- | --- | --- | --- | --- | --- |
|  | Representativeness | Selection | Ascertainment | Demonstration | Control for Confounders | Assessment | Follow-up period | Adequacy of follow up |  |
| Cheng et al., 2022 | 0 | 0 | 0 | 0 | 1 | 1 | 1 | 1 | 4 |
| Lai et al., 2018 | 1 | 1 | 1 | 0 | 2 | 1 | 1 | 1 | 8 |
| Li et al., 2019 | 0 | 1 | 1 | 0 | 1 | 1 | 1 | 1 | 6 |
| Liu et al., 2022 | 1 | 1 | 1 | 0 | 1 | 1 | 1 | 1 | 7 |
| Looman et al., 2019 | 1 | 1 | 1 | 1 | 1 | 1 | 1 | 1 | 8 |
| Saravanan et al., 2021 | 1 | 1 | 1 | 1 | 2 | 1 | 1 | 1 | 9 |
| Van Weelden et al., 2021 | 1 | 1 | 1 | 1 | 2 | 1 | 1 | 1 | 9 |
| Wang et al., 2022 (a) | 1 | 1 | 1 | 1 | 1 | 1 | 1 | 1 | 8 |
| Wang et al., 2023 (b) | 0 | 1 | 1 | 0 | 1 | 1 | 1 | 1 | 6 |
| Zhang et al., 2024 | 1 | 1 | 1 | 0 | 1 | 1 | 1 | 1 | 7 |

## Supplementary Table 4: Data Transformations used in the Meta-analysis

| **Author, Year** | **Serum folate** | **Original reported effect size** | **Transformed effect size and steps used to derive the estimate** |
| --- | --- | --- | --- |
| Lai et al., 2017 | Pooled serum folate median (IQR): 45.0 (30.9, 58.1) nmol/L for the combined sample of 913 women (Modified by weighted pooling of median then converted by 1.3 of plasma levels) | Unadjusted model: β = -0.03 (95% CI: -0.06, 0.001), p = 0.056 Model 1 (adjusted for maternal factors): β = -0.02 (95% CI: -0.06, 0.01), p = 0.124 Model 2 (adjusted for maternal factors and other B vitamins): β = -0.02 (95% CI: -0.05, 0.02), p = 0.358 | Step 1: Back transform the std beta to unstandardized beta  Step 2: Take anti-log of the unstandardized beta; Then calculate the std. beta for SD change  FPG: Unadjusted model: -0.001 mmol/L Model 1: -0.001 mmol/L Model 2: -0.001 mmol/L  2-h Glucose: Unadjusted model: 0.0074mmol/L Model 1: 0.0049 mmol/L Model 2: 0.0043 mmol/L |
| Li et al., 2019 | The pooled serum folate median (IQR) in nmol/L is: 20.4 (13.8, 31.7) nmol/L for the combined sample of 406 women (Modified by weighted pooling of median of serum folate) | Folate and FPG: Crude model: β = 0.04 (95% CI: -0.03, 0.11), p = 0.258 Model 1 (adjusted for maternal factors): β = 0.05 (95% CI: -0.02, 0.13), p = 0.157 Model 2 (adjusted for maternal factors and other B vitamins): β = 0.08 (95% CI: 0.01, 0.16), p = 0.044 Folate and 1-h Plasma Glucose: Crude model: β = 0.60 (95% CI: 0.30, 0.91), p < 0.001 Model 1 (adjusted for maternal factors): β = 0.49 (95% CI: 0.19, 0.80), p = 0.002 Model 2 (adjusted for maternal factors and other B vitamins): β = 0.51 (95% CI: 0.19, 0.83), p = 0.002 Folate and 2-h Plasma Glucose: Crude model: β = 0.46 (95% CI: 0.22, 0.69), p < 0.001 Model 1 (adjusted for maternal factors): β = 0.44 (95% CI: 0.19, 0.68), p = 0.001 Model 2 (adjusted for maternal factors and other B vitamins): β = 0.45 (95% CI: 0.19, 0.71), p = 0.001 | Step 1: Convert the log transformed coefficient into absolute coefficient  Step 2: Standardize the absolute coefficient to std beta using SD (x)/SD (y) * beta  FPG: Crude model: 0.0019 mmol/L Model 1: 0.0024 mmol/L Model 2: 0.0038 mmol/L  1-h plasma glucose: Crude model: 0.0288 mmol/L Model 1: 0.0235 mmol/L Model 2: 0.0245 mmol/L  2-h plasma glucose: Crude model: 0.0221 mmol/L Model 1: 0.0211 mmol/L Model 2: 0.0216 mmol/L |
| Liu et al., 2021 | Mean serum folate (SD): 42.29 (12.7) nmol/L in 42478 women | Crude model (all women): Fasting plasma glucose: β = 0.00 (95% CI: -0.00, 0.01), not significant 1-hour plasma glucose: β = 0.17 (95% CI: 0.15, 0.18), p < 0.001 2-hour plasma glucose: β = 0.14 (95% CI: 0.12, 0.15), p < 0.001  Adjusted model (adjusted for BMI status, fetal gender, vitamin B12, parity, maternal age and education): Fasting plasma glucose: β = 0.01 (95% CI: 0.00, 0.01), p < 0.05 1-hour plasma glucose: β = 0.15 (95% CI: 0.13, 0.17), p < 0.001 2-hour plasma glucose: β = 0.12 (95% CI: 0.11, 0.13), p < 0.001 | No transformations were needed to include this paper in this quantitative analysis |
| Looman et al., 2018 | Converted serum folate Mean (SD): 53.4 (2.7) nmol/L in 105 women (Modified by 1.3 of plasma levels) | Fasting glucose: Unstandardized regression coefficient (β): -0.003 mmol/L; 95% CI: (-0.007, 0.002); p-value: 0.261 2-hour glucose: Unstandardized regression coefficient (β): -0.002 mmol/L; 95% CI: (-0.013, 0.008); p-value: 0.656 | Unable to get the standardized regression coefficient due to the lack of SD(Y) data for the glucose measured, therefore this study cannot be included in the meta-analysis |
| Wang et al., 2022 (a) | Mean serum folate Median (IQR): 11.8 (10.1–13.9) in 1065 women | Correlation with fasting glucose: r = -0.010 (not statistically significant) Correlation with 1-hour glucose: r = 0.025 (not statistically significant) Correlation with 2-hour glucose: r = 0.012 (not statistically significant) | β = r This is because a standardized regression coefficient from a simple linear regression with a single predictor is equal to the Pearson correlation coefficient between the predictor and outcome variables. |
| Saravanan et al., 2021 | Serum folate Median (IQR): 35.9 (24.8- 52.2) in 4320 women | Fasting glucose: Model 1: β = 0.018 (95% CI: 0.0014, 0.0346) Model 2: β = 0.0079 (95% CI: -0.0083, 0.0241) 2-hour glucose: Model 1: β = 0.0822 (95% CI: 0.0363, 0.1281) Model 2: β = 0.0678 (95% CI: 0.0221, 0.1135) | No transformations were needed to include this paper in this quantitative analysis |
| van Weelden et al., 2020 | Serum folate Mean (SD): 9.6 (4.9) in 959 women | Fasting Glucose Unstandardized Coefficients Unadjusted: β = 0.002 mmol/L; 95% CI: (-0.005, 0.009); p-value: 0.58 Model 1: β = 0.0005 mmol/L; 95% CI: (-0.007, 0.008); p-value: 0.90 Model 2: β = -0.003 mmol/L; 95% CI: (-0.008, 0.007); p-value: 0.93 Model 3: β = 0.0007 mmol/L; 95% CI: (-0.007, 0.008); p-value: 0.85  1-Hour Glucose Unadjusted: β = 0.046 mmol/L; 95% CI: (0.018, 0.074); p-value: 0.001 Model 1: β = 0.033 mmol/L; 95% CI: (0.003, 0.063); p-value: 0.032 Model 2: β = 0.029 mmol/L; 95% CI: (-0.001, 0.059); p-value: 0.055 Model 3: β = 0.031 mmol/L; 95% CI: (0.001, 0.061); p-value: 0.045  2-Hour Glucose Unadjusted: β = 0.017 mmol/L; 95% CI: (-0.003, 0.037); p-value: 0.09 Model 1: β = 0.011 mmol/L; 95% CI: (-0.010, 0.033); p-value: 0.29 Model 2: β = 0.009 mmol/L; 95% CI: (-0.012, 0.030); p-value: 0.39 Model 3: β = 0.011 mmol/L; 95% CI: (-0.010, 0.033); p-value: 0.30 | Using the formula from Nienmann et al for standardization with β = (SD(X) / SD(Y)) * b formula:  Fasting Glucose: Unadjusted: β = 0.037 mmol/L  Model 1: β = 0.009 mmol/L Model 2: β = -0.056 mmol/L Model 3: β = 0.013 mmol/L  1-Hour Glucose: Unadjusted: β = 0.244 mmol/L  Model 1: β = 0.175 mmol/L Model 2: β = 0.154 mmol/L Model 3: β = 0.164 mmol/L  2-Hour Glucose: Unadjusted: β = 0.126 mmol/L  Model 1: β = 0.082 mmol/L Model 2: β = 0.067 mmol/L Model 3: β = 0.082 mmol/L |
| Wang et al., 2023 (b) | The pooled serum folate median (IQR) is: 9.3 (6.1, 14.4) in 1254 women (Modified by weighted pooling of median of serum folate) | Fasting Plasma Glucose: Unadjusted: β = 0.05 mmol/L; 95% CI: (0.01, 0.09); p-value: 0.023 Adjusted Model: β = 0.07 mmol/L; 95% CI: (0.03, 0.12); p-value: 0.002 1-Hour Plasma Glucose (1-h PG): Unadjusted: β = 0.44 mmol/L; 95% CI: (0.29, 0.59); p-value: <0.001 Adjusted Model: β = 0.32 mmol/L; 95% CI: (0.16, 0.48); p-value: <0.001 2-Hour Plasma Glucose (2-h PG): Unadjusted: β = 0.35 mmol/L; 95% CI: (0.23, 0.47); p-value: <0.001 Adjusted Model: β = 0.29 mmol/L; 95% CI: (0.15, 0.42); p-value: <0.001 These coefficients are for per interquartile range (IQR) increase in folate and are adjusted for age, prepregnancy BMI, education, smoking, drinking, family history of diabetes, parity, and rs1801131 genotypes. | No transformations were needed to include this paper in this quantitative analysis |

## Supplementary Figure 1: Association between maternal folate and fasting glucose by pooling unadjusted regression coefficients


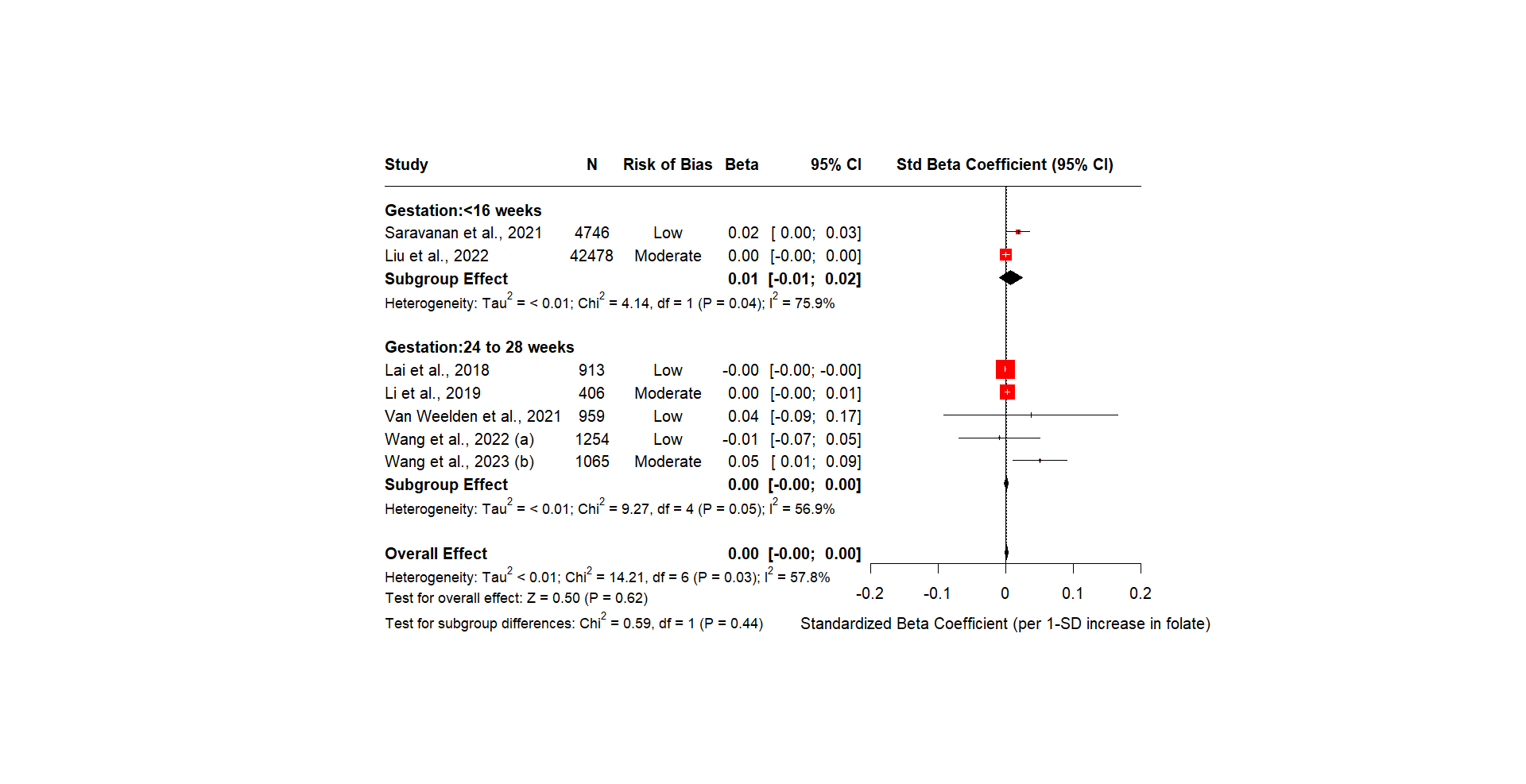


Standardised β-coefficients (Std. βeta) and 95% confidence interval (CI) of fasting glucose for absolute 1 standard deviation (SD)., 1 nmol/l change in maternal serum folate including early pregnancy and mid-pregnancy circulating folate concentrations.

## Supplementary Figure 2: Association between maternal folate and 1-hr post glucose load by pooling unadjusted regression coefficients


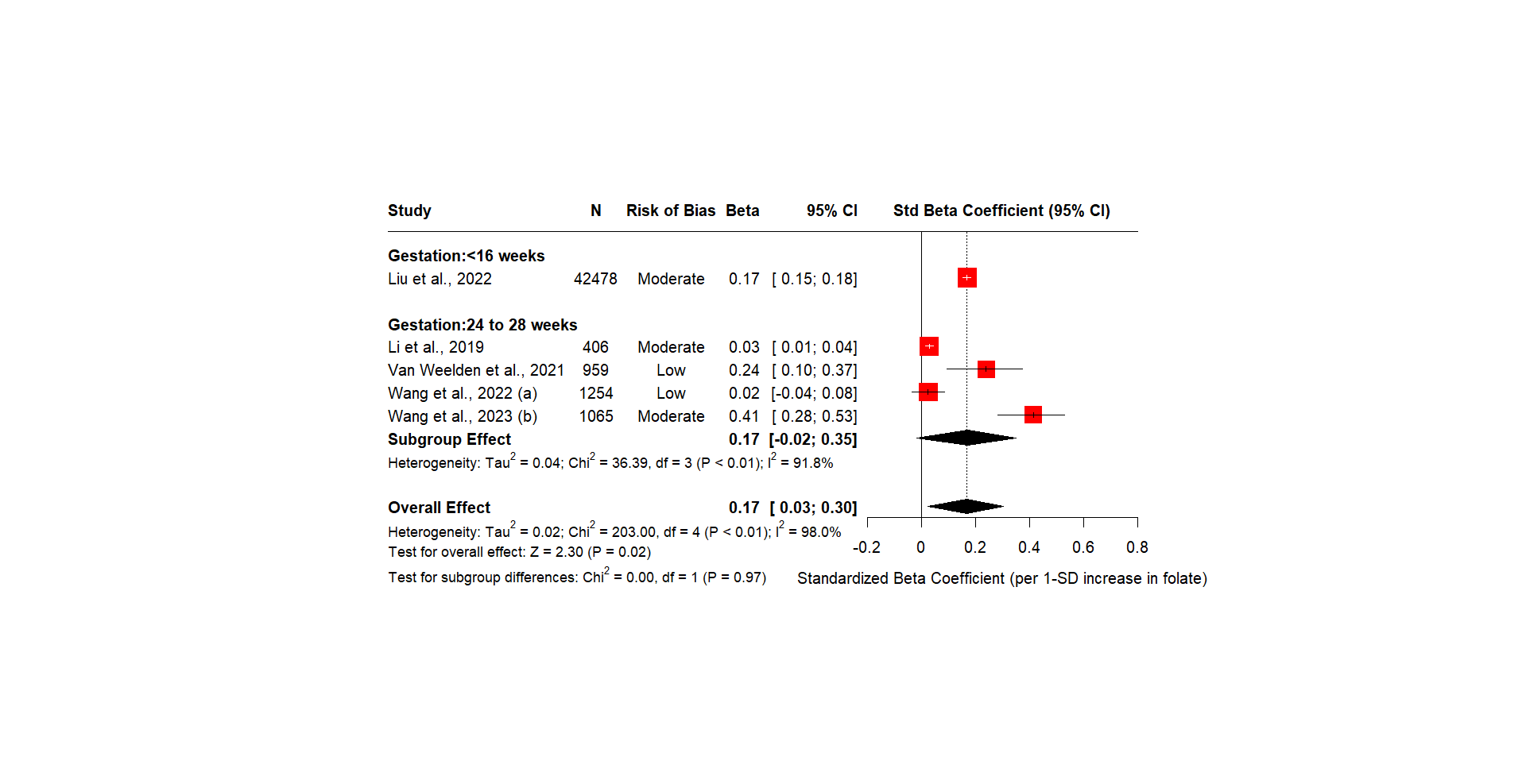


Standardised β-coefficients (Std. βeta) and 95% confidence interval (CI) of 1-hr post load glucose for absolute 1 standard deviation (SD)., 1 nmol/l change in maternal serum folate including early pregnancy and mid-pregnancy circulating folate concentrations.

## Supplementary Figure 3: Association between maternal folate and 2-hr post glucose load by pooling unadjusted regression coefficients


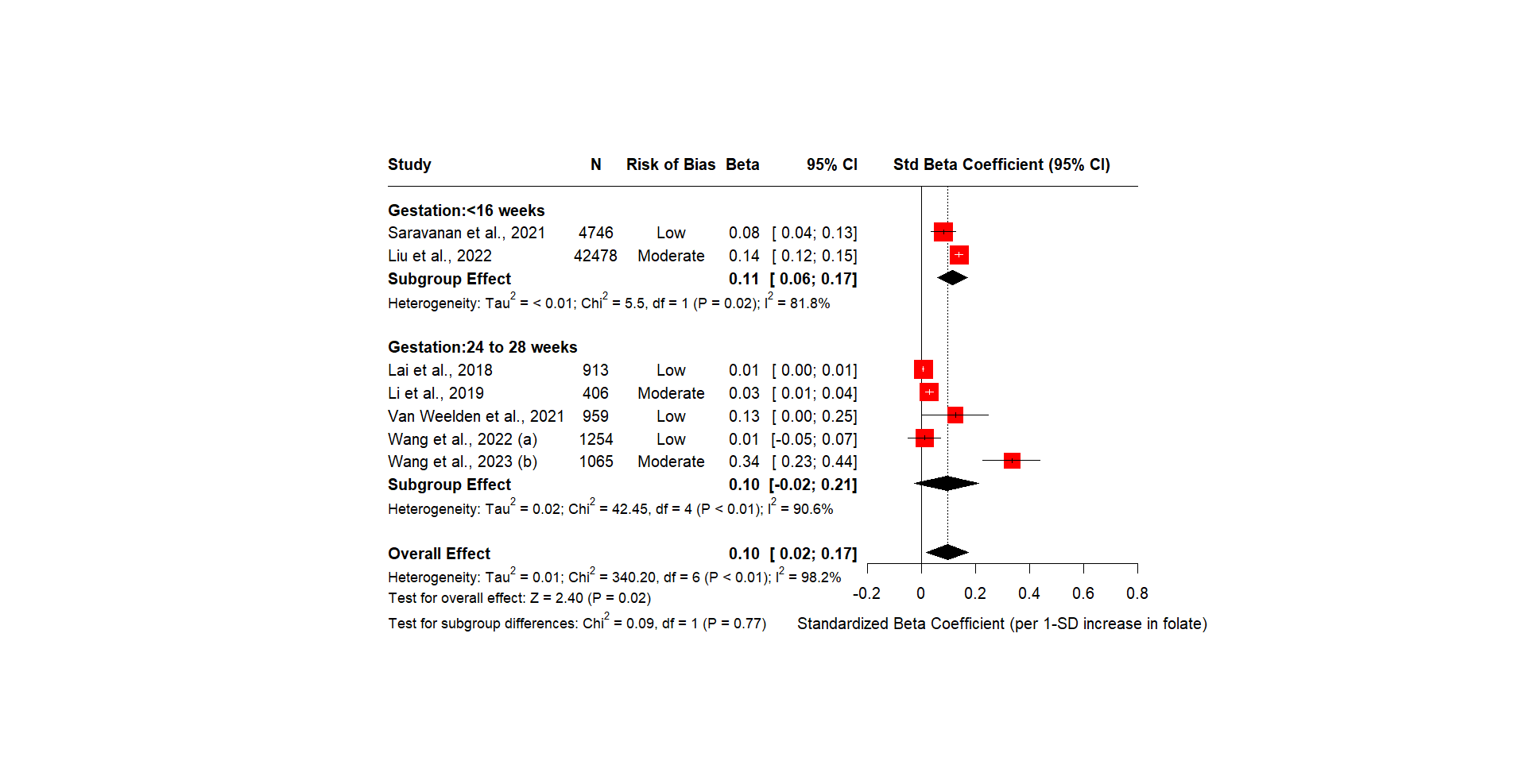


Standardised β-coefficients (Std. βeta) and 95% confidence interval (CI) of 2-hr post load glucose for absolute 1 standard deviation (SD)., 1 nmol/L change in maternal serum folate including early pregnancy and mid-pregnancy circulating folate concentrations.

## Supplementary Figure 4: Leave one out sensitivity analysis of studies reported maternal folate and fasting glucose


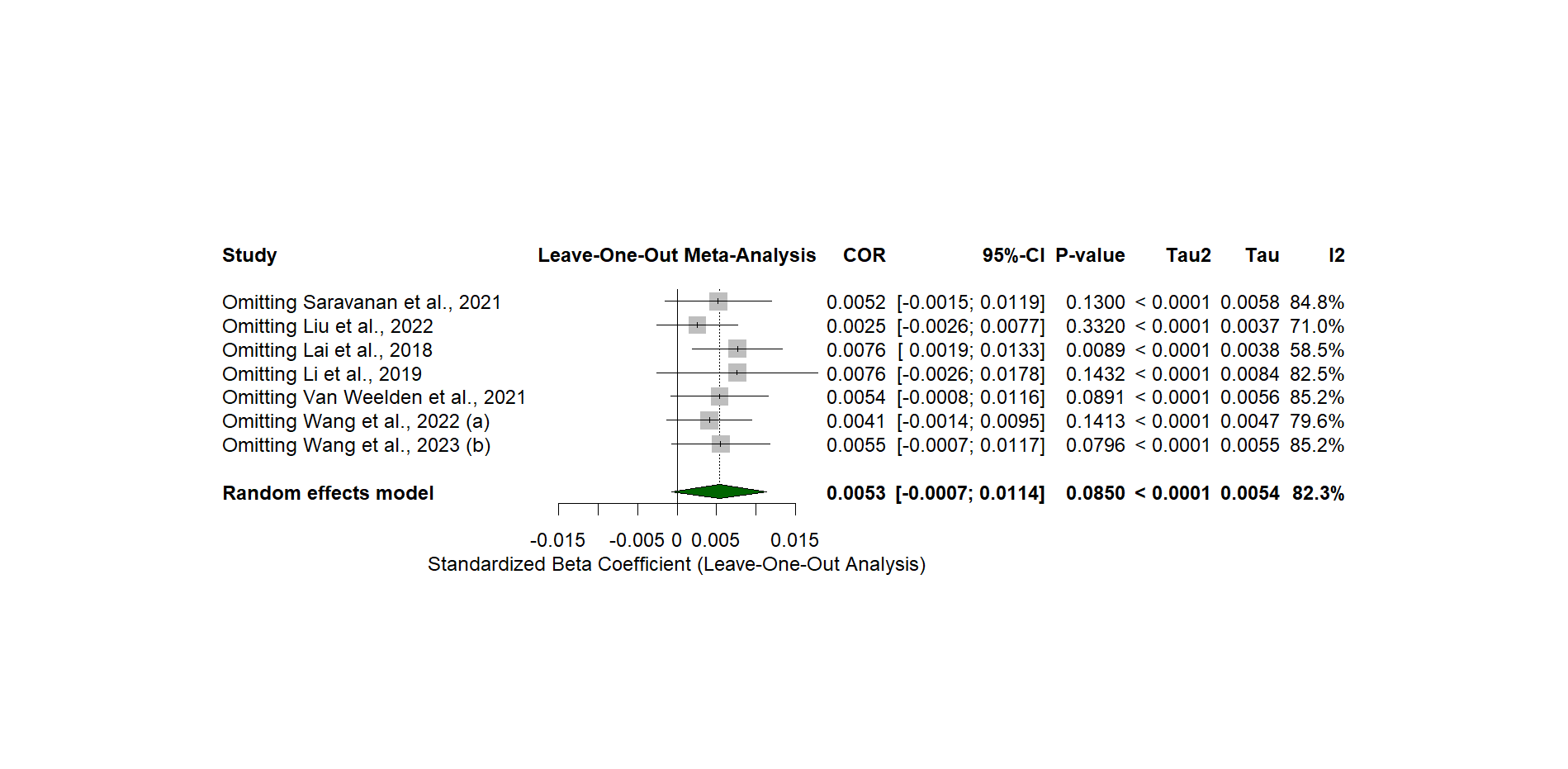


Leave-one-out sensitivity analysis for the association between maternal folate and fasting plasma glucose levels. Each point represents the pooled effect estimate when one study is sequentially omitted, demonstrating the robustness of the overall meta-analysis results across different study combinations.

## Supplementary Figure 5: Funnel plot to assess the publication between studies reported maternal folate and fasting glucose

##
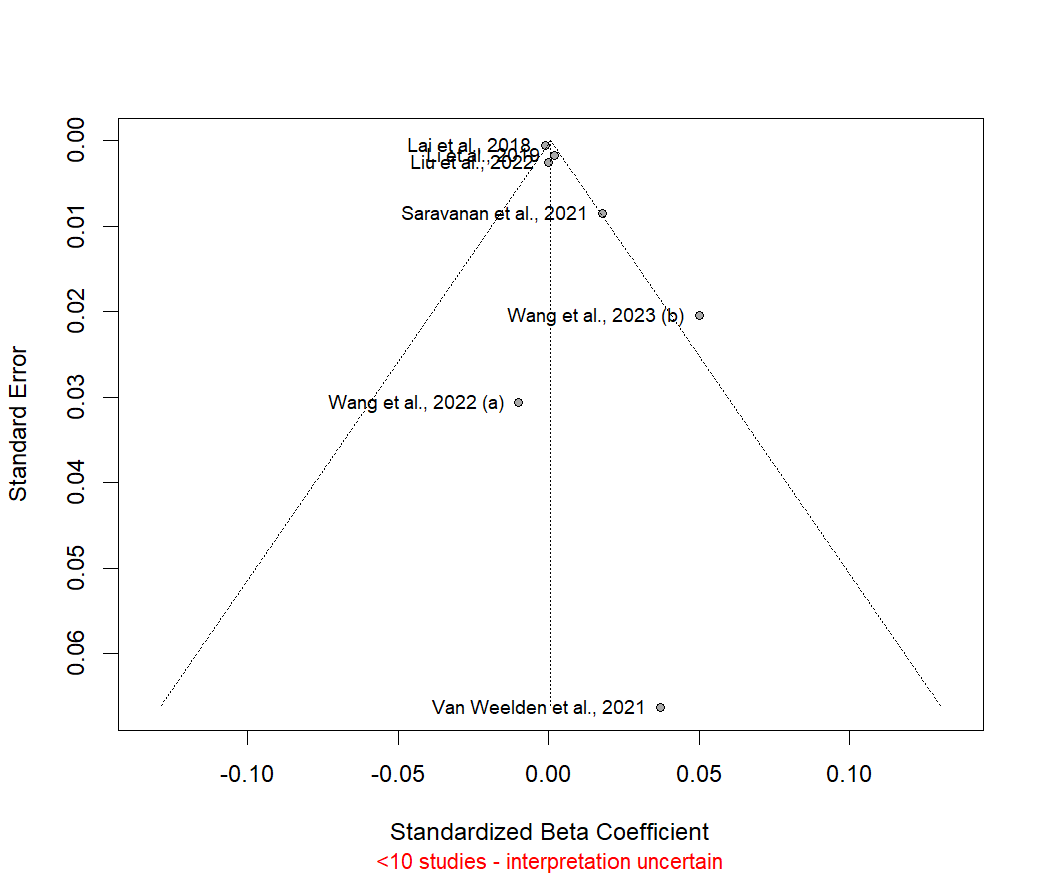


Funnel plot assessing publication bias in studies reporting the association between maternal circulating folate and fasting plasma glucose. The vertical line represents the combined effect size, while the dashed lines illustrate the 95% confidence intervals.

**Supplementary Figure 6: Leave one out sensitivity analysis of studies reported maternal folate and 1-hr glucose**


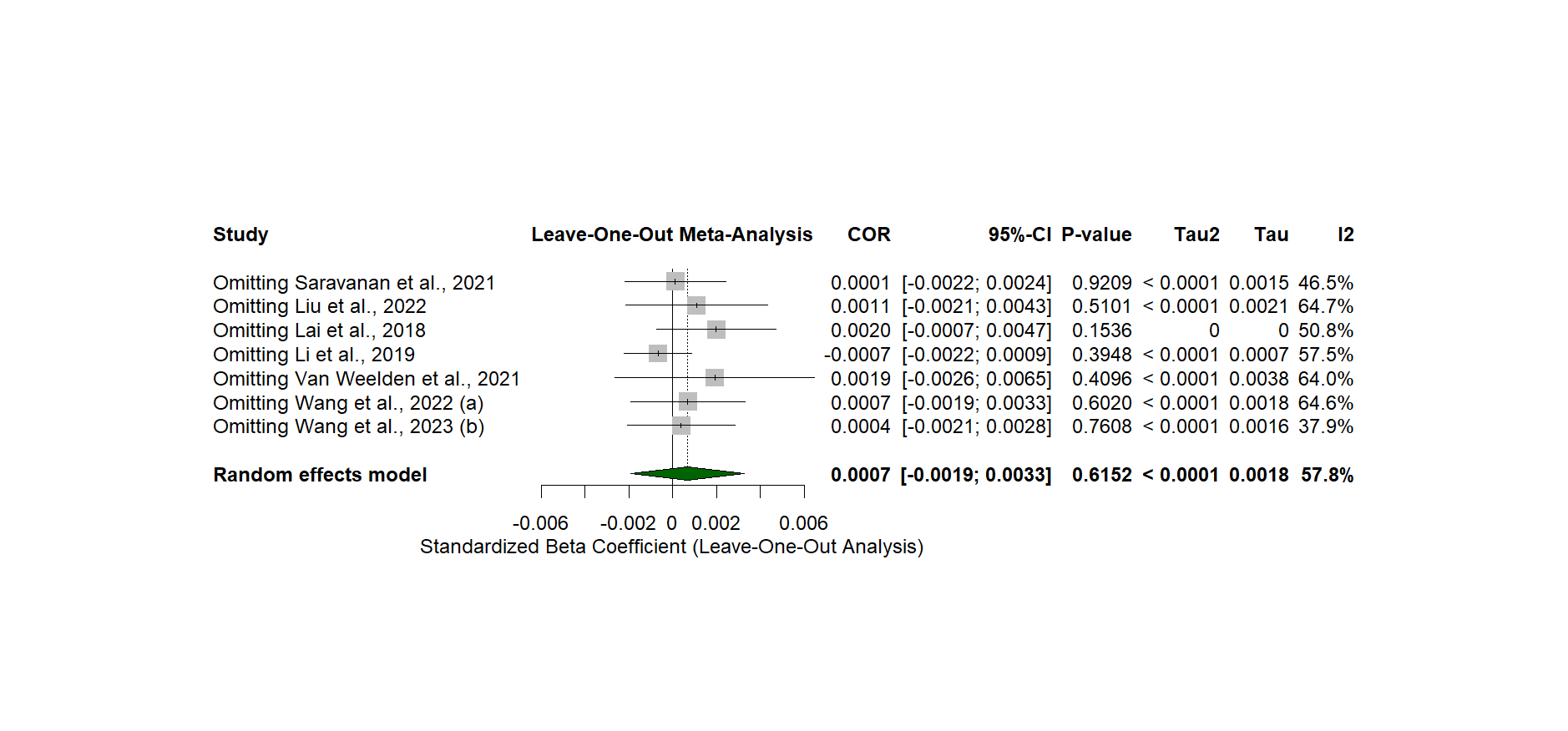


Leave-one-out sensitivity analysis for the association between maternal folate and 1-hr plasma glucose levels. Each point represents the pooled effect estimate when one study is sequentially omitted, demonstrating the robustness of the overall meta-analysis results across different study combinations.

**Supplementary Figure 7: Funnel plot to assess the publication between studies reported maternal folate and 1-hr glucose**

**
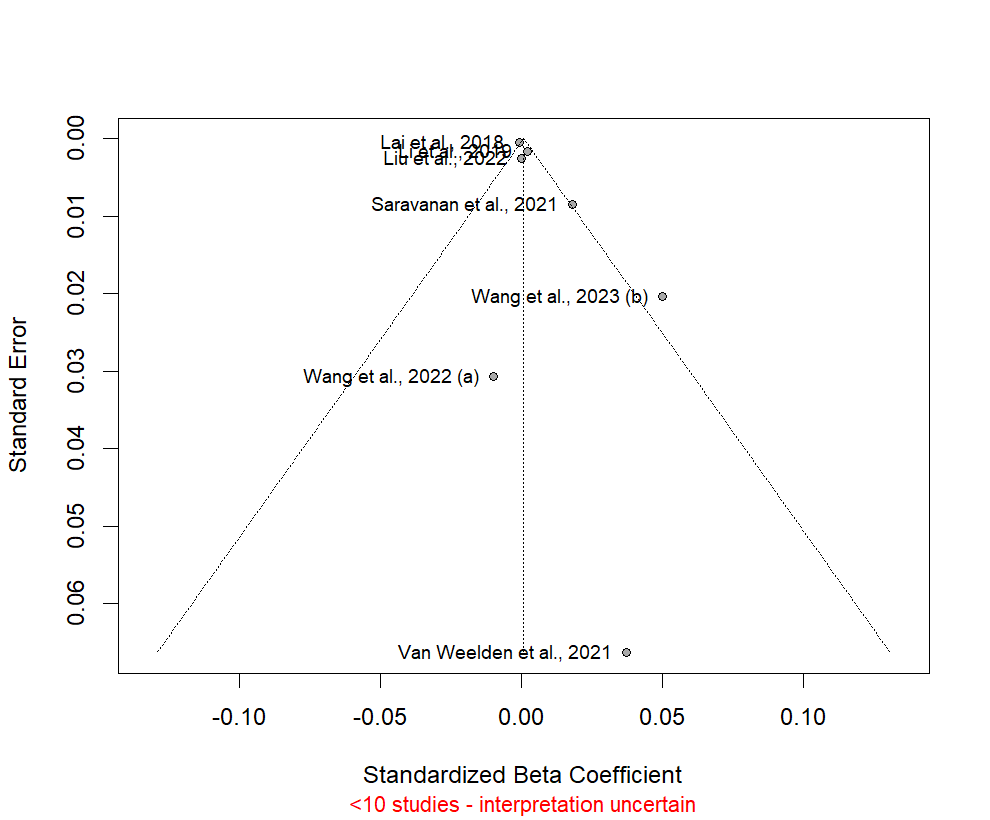
**

Funnel plot assessing publication bias in studies reporting the association between maternal circulating folate and 1-hr plasma glucose. The vertical line represents the combined effect size, while the dashed lines illustrate the 95% confidence intervals

**Supplementary Figure 8: Leave one out sensitivity analysis of studies reported maternal folate and 2-hr glucose**

**
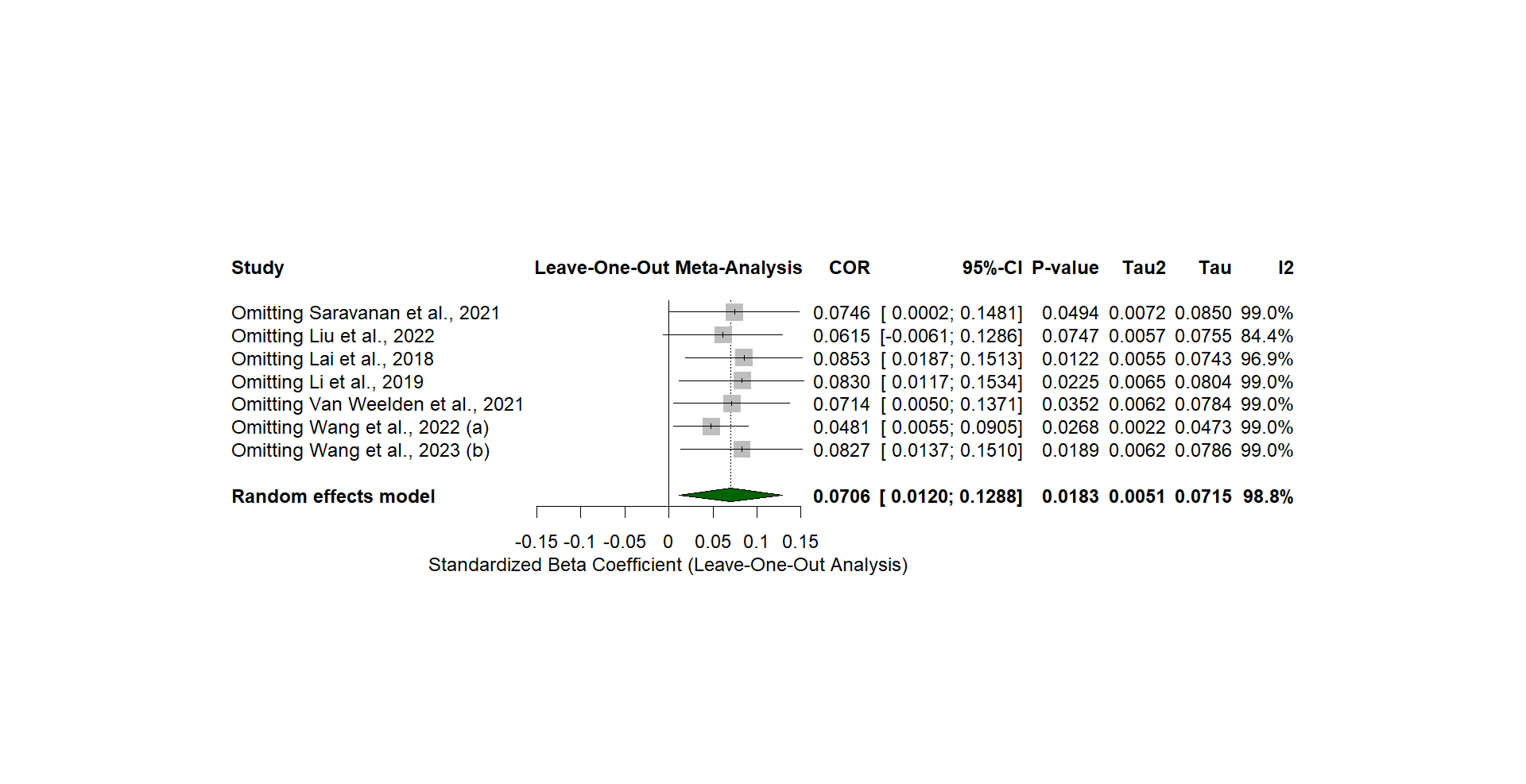
**

Leave-one-out sensitivity analysis for the association between maternal folate and 2-hr plasma glucose levels. Each point represents the pooled effect estimate when one study is sequentially omitted, demonstrating the robustness of the overall meta-analysis results across different study combinations.

## Supplementary Figure 9: Funnel plot to assess the publication between studies reported maternal folate and 2-hour glucose

**
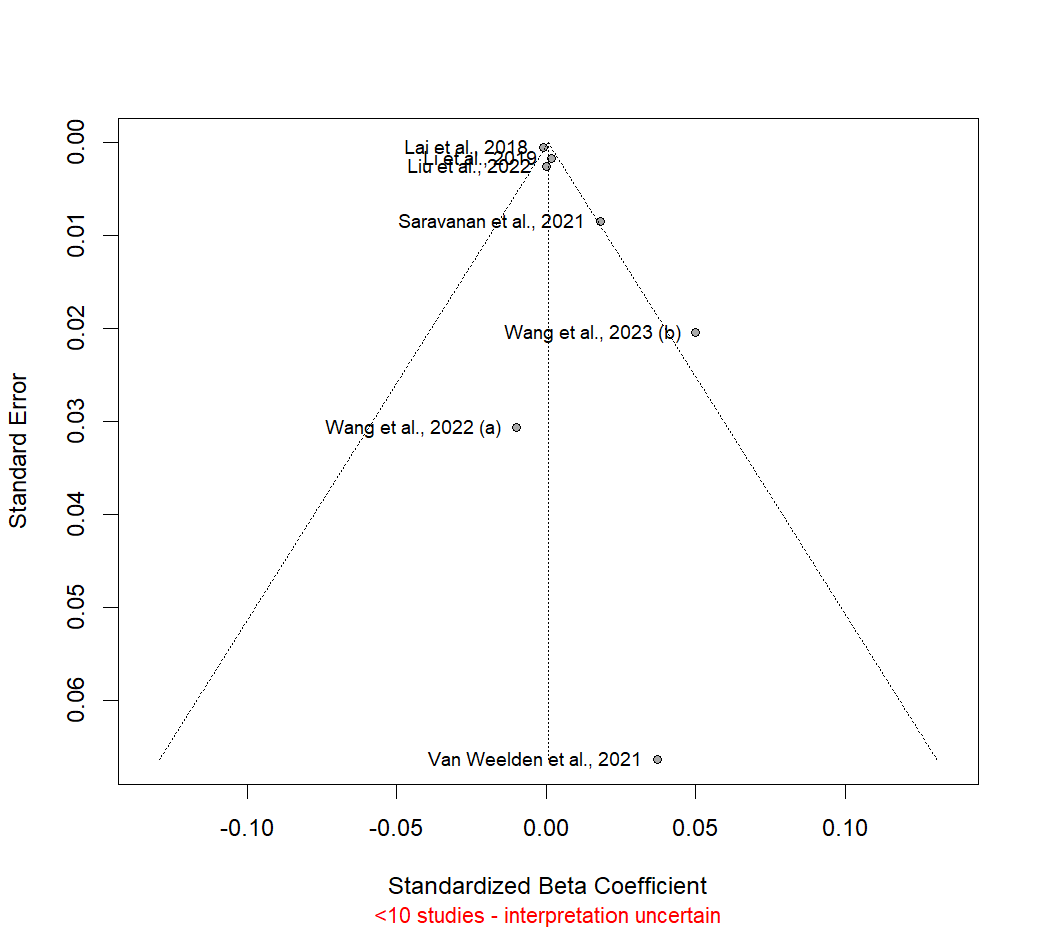
**

Funnel plot assessing publication bias in studies reporting the association between maternal circulating folate and 2-hr plasma glucose. The vertical line represents the combined effect size, while the dashed lines illustrate the 95% confidence intervals.
